# Supplementary material for: T3 Intratracheal Therapy Alleviates Pulmonary Pathology in an Elastase-Induced Emphysema-Dominant COPD Mouse Model
Source: Antioxidants (Basel). 2023 Dec 22;13(1):30. doi: 10.3390/antiox13010030 (PMC10812479; doi:10.3390/antiox13010030)
Supplement: Supplementary file 1 [file antioxidants-13-00030-s001.zip › antioxidants-2693767-supplementary.pdf]

antioxidants

Article

T_3 intratracheal therapy alleviates pulmonary pathology in an elastase-induced emphysema-dominant COPD mouse model

Noriki Takahashi 1,2, Ryunosuke Nakashima 1, Aoi Nasu 1,2 Megumi Hayashi 1 Haruka Fujikawa 1,2, Taisei Kawakami 1, Yuka Eto 1, Tomoki Kishimoto 1, Ayami Fukuyama 1, Choyo Ogasawara 1, Keisuke Kawano 1, Yukio Fujiwara4, Mary Ann Suico 1,3, Hirofumi Kai 1,3 and Tsuyoshi Shuto 1,3,*

1 Department of Molecular Medicine, Graduate School of Pharmaceutical Sciences, Kumamoto University, 5-1 Oe-honmachi, Chuo-ku, Kumamoto 862-0973, Japan; noriki.t07@outlook.jp (N.T.); 158y1012@st.kumamoto-u.ac.jp (H.F.); 137p1015@st.kumamoto-u.ac.jp (T.K.); 161p2023@st.kumamoto-u.ac.jp (A.N.); 212y1010@st.kumamoto-u.ac.jp (M.H.); 181p1015@st.kumamoto-u.ac.jp (T.K.); 191p1050@st.kumamoto-u.ac.jp (A.F.); 191p2005@st.kumamoto-u.ac.jp (C.O.); 207p2006@st.kumamoto-u.ac.jp (K.K.); fuji-y@kumamoto-u.ac.jp (Y.F.); mann@gpo.kumamoto-u.ac.jp (M.A.S); hirokai@gpo.kumamoto-u.ac.jp (H.K.)

2 Program for Leading Graduate Schools "HIGO (Health life science: Interdisciplinary and Global Oriented) Program", Graduate School of Pharmaceutical Sciences, Kumamoto University, 5-1 Oe-honmachi, Chuo-ku, Kumamoto 862-0973, Japan

3 Global Center for Natural Resources Sciences, Faculty of Life Sciences, Kumamoto University, 5-1 Oe-Honmachi, Chuo-ku, Kumamoto, 862-0973, Japan

4 Department of Cell Pathology, Graduate School of Medical Sciences, Kumamoto University, 1-1-1, Honjo, Kumamoto Chuo-ku, Kumamoto, 860-8556, Japan

* Correspondence: tshuto@gpo.kumamoto-u.ac.jp (T.S.); Tel.: +81-96-371-4407 (T.S.)

Supplementary figures

Supplementary Figure S1

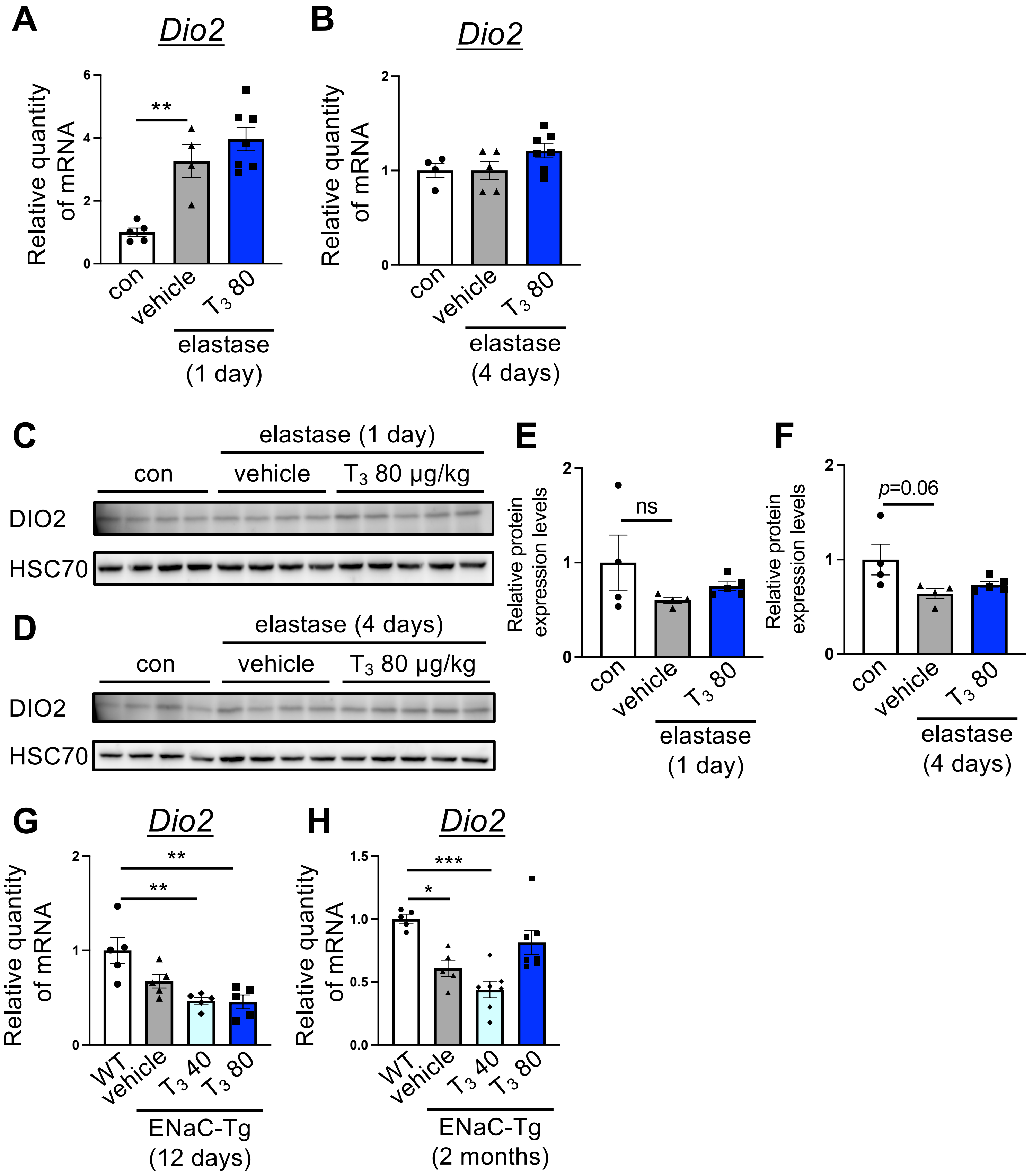

Supplementary Figure S1 The change in T_3 requirement by the intratracheal administration of T_3. (**A-B, G-H**) The relative quantity of mRNA levels of *Dio2* were measured by RT-qPCR in the lungs of control, elastase-treated and intratracheally T_3-administrated mice (80 µg/kg, one injection or every other day for 4 days after 1 day of elastase treatment (**A, B**)) or WT or C57BL/6J-βENaC-Tg mice and intratracheally T_3-administrated C57BL/6J-βENaC-Tg mice (40 or 80 µg/kg, every other day for 12 days or 2 months at 13-weeks-old (**G, H**)). (**C-F**) The protein expression levels of DIO2 in the lung of control, elastase-treated and intratracheally T_3-administrated mice (80 µg/kg, one injection (**C, E**) or every other day for 4 days (**D, F**) after 1 day of elastase treatment) by immunoblotting. HSC70 was used as the loading control. The band intensity was quantified by Multi Gauge software (FUJIFILM, Japan).

Data are means ± S.E.M.; *n*=4-7 mice/group. P values were assessed by ANOVA with Tukey-Kramer procedure. *$p<0.05$, **$p<0.01$, *** $p<0.001$ (vs. control or WT), ns: not significant

Supplementary Figure S2

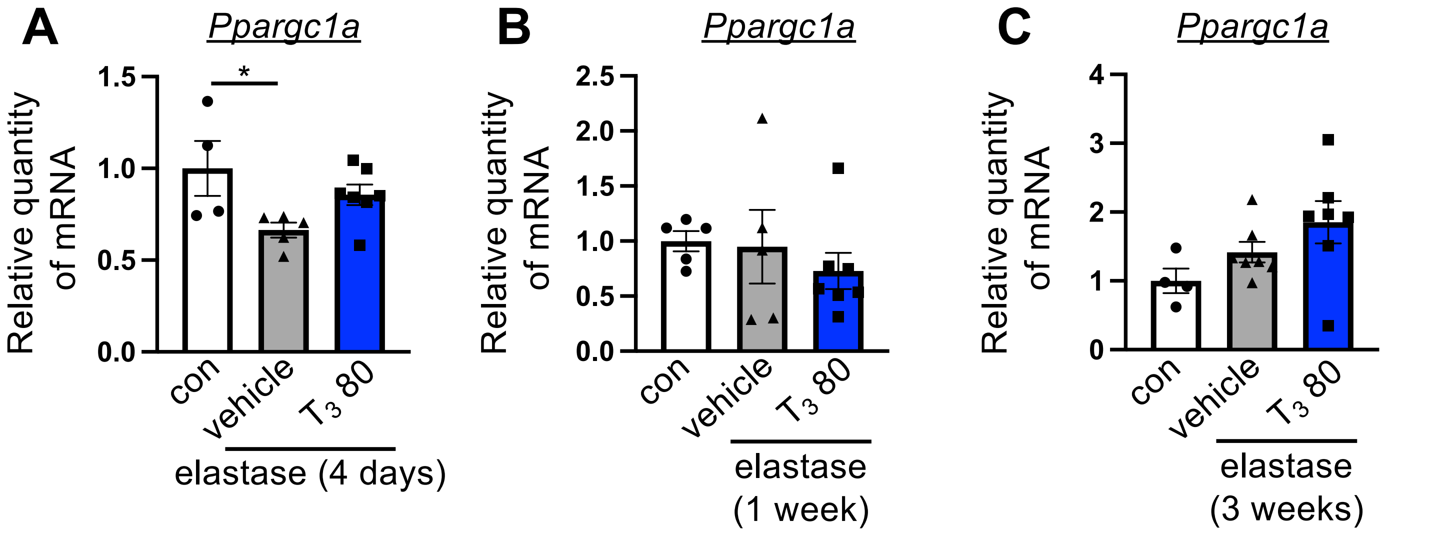

Supplementary Figure S2 The effect of intratracheal administration of T_3 on mitochondrial function in elastase-induced COPD model mice. (**A-C**)The relative quantity of mRNA levels of *Ppargc1a* was measured, by RT-qPCR, in the lungs of control (n=4-5), elastase-treated (n=5-7) and intratracheally T_3-administrated (n=5-7) mice (80 μg/kg, every other day for 4 days, 1 week or 3 weeks, 1 day after elastase treatment).
Data are means ± S.E.M.; P values were assessed by ANOVA with Tukey-Kramer procedure. *p<0.05, (vs. control)

Supplementary Figure S3

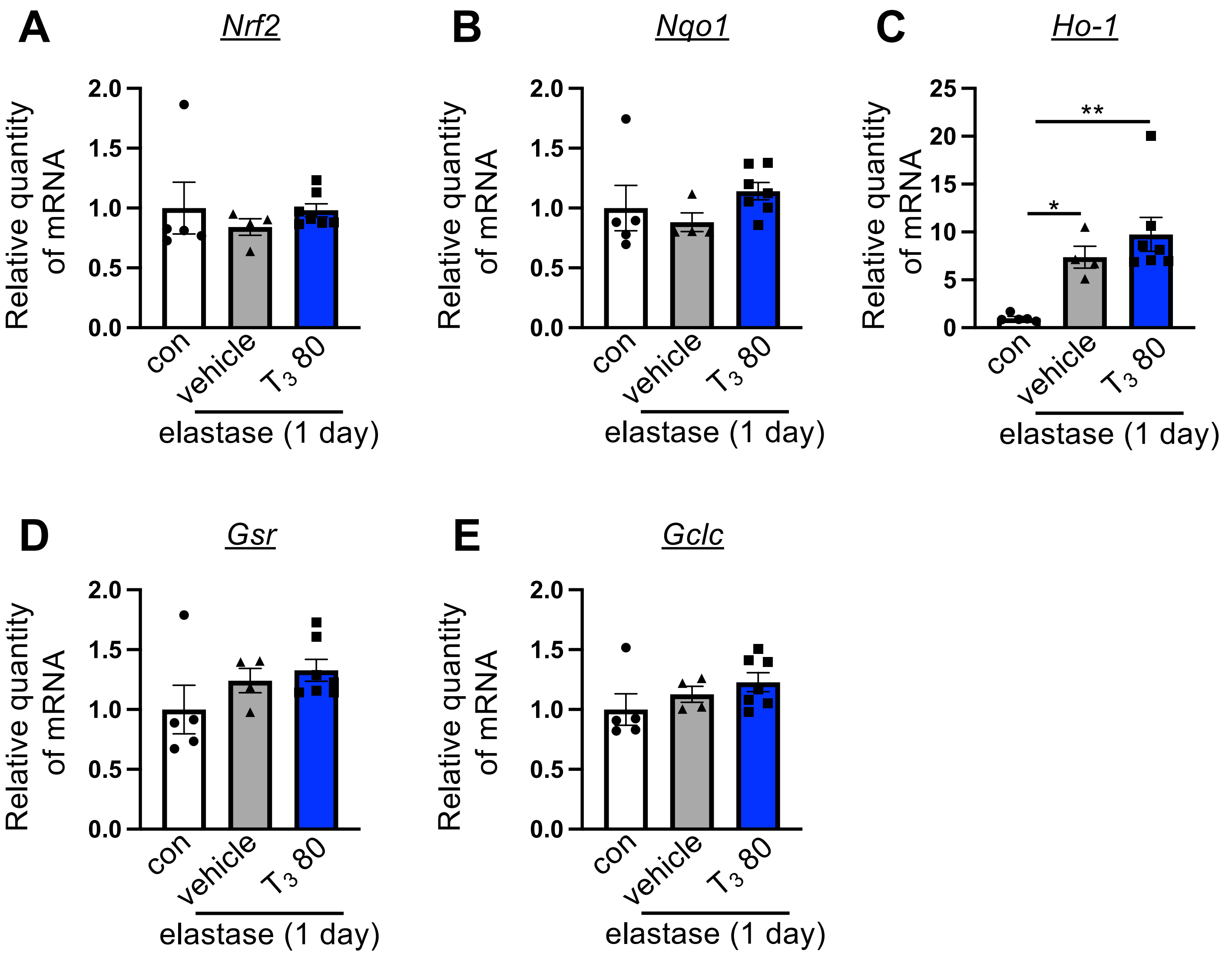

Supplementary Figure S3 The effect of intratracheal administration of T_3 on the expression of oxidative stress-related factors in elastase-induced COPD model mice. (**A-E**) The relative quantity of mRNA levels of the indicated genes were measured in the lungs of control (n=5), elastase-treated (n=4) and intratracheally T_3-administrated (n=7) mice (80 μg/kg, one injection the day after elastase treatment).

Data are means ± S.E.M.; P values were assessed by ANOVA with Tukey-Kramer procedure. *$p<0.05$, **$p<0.01$, (vs. control)

Supplementary Figure S4

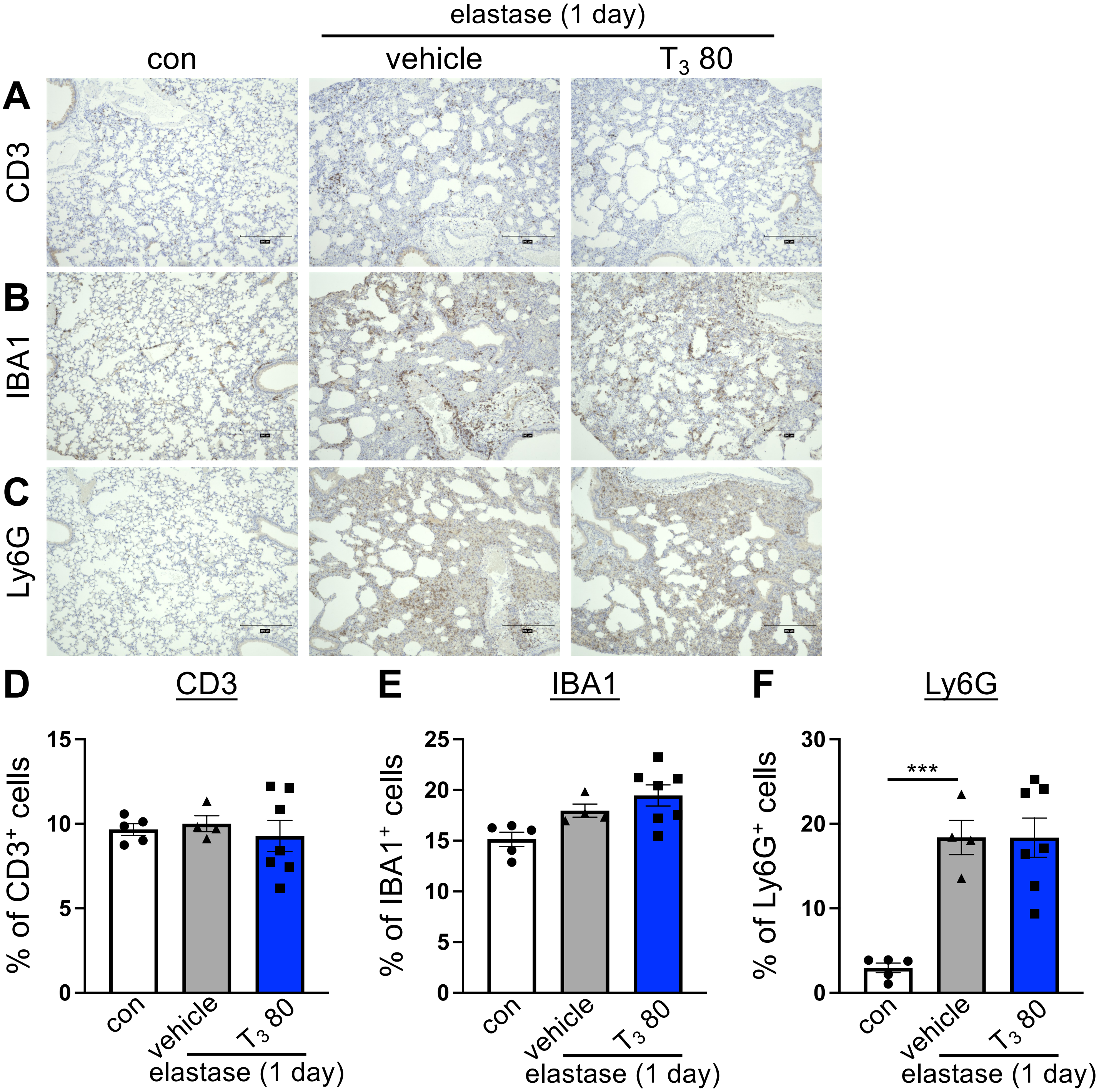

Supplementary Figure S4 The effect of intratracheal administration of T_3 on the infiltration of immune cells into lung tissues in elastase-induced COPD model mice. (**A-C**) Immunohistochemistry of CD3 (T cell marker) (**A**), IBA1 (macrophage marker) (**B**), Ly6G (neutrophil marker) (**C**) in the lungs of control, elastase-treated and intratracheally T_3-administrated mice (80 µg/kg, one injection the day after elastase treatment). Scale bar = 300 µm. (**D-F**) Percentages of CD3 (**D**), IBA1 (**E**) or Ly6G (**F**) positive cells in total cells were showed in the lungs of representative mice. These data were quantified by HALO, image analysis software.

Data are means ± S.E.M.; *n*=4-7 mice/group. P values were assessed by ANOVA with Tukey-Kramer procedure.

*** *p*<0.001 (vs. control)

Supplementary Figure S5

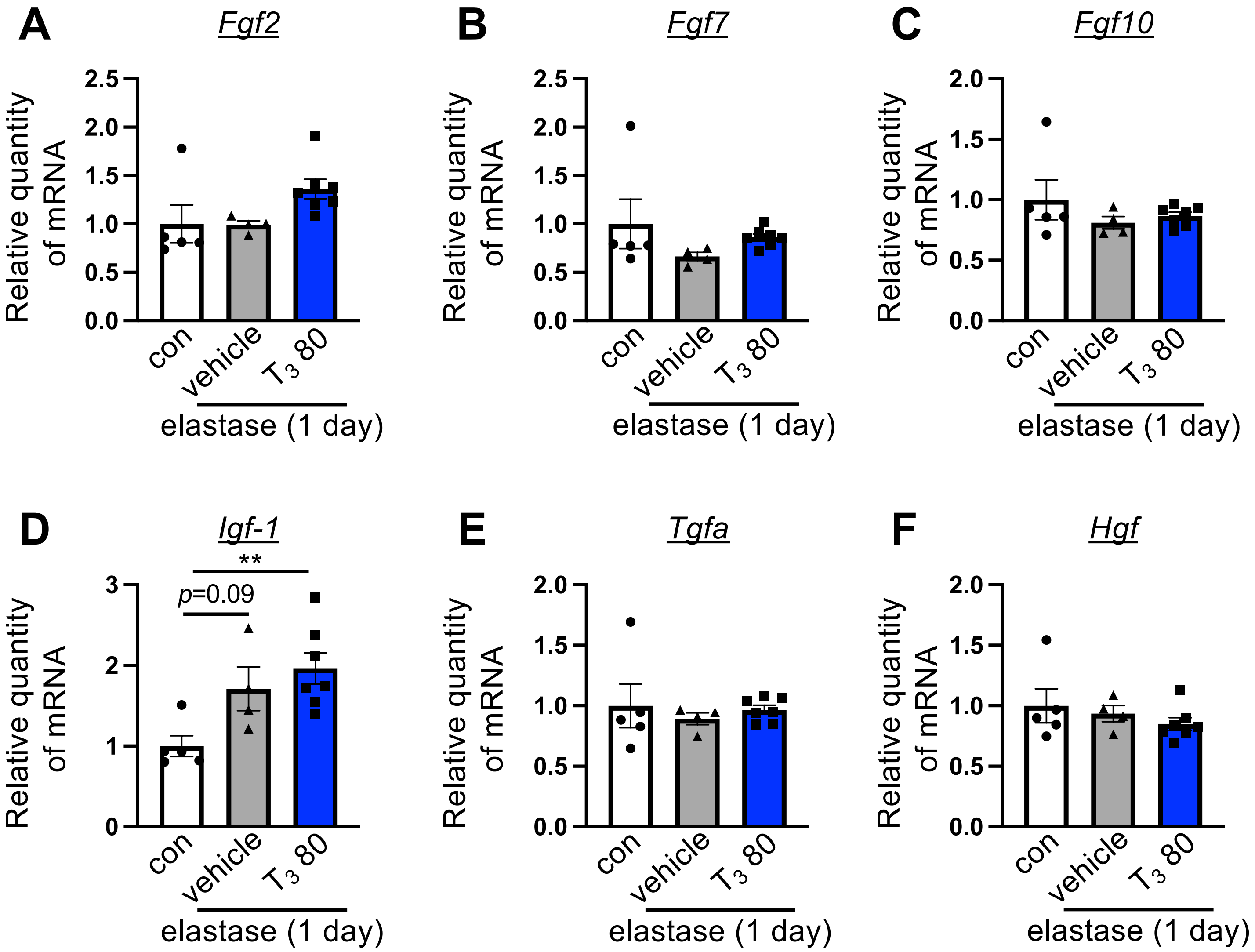

Supplementary Figure S5 The effect of intratracheal administration of T_3 on the expression of growth factors in elastase-induced COPD model mice. (**A-F**) The relative quantity of mRNA levels of *Fgf2, Fgf7, Fgf10, Igf-1, Tgfa* and *Hgf* were measured in the lungs of control (n=5), elastase-treated (n=4) and intratracheally T_3-administrated (n=7) mice (80 μg/kg, one injection the day after elastase treatment).
Data are means ± S.E.M.; P values were assessed by ANOVA with Tukey-Kramer procedure. ***p*<0.01, (vs. control)
